# Functional Limitations in Heart Failure and Cancer: An Analysis of the Medicare Health Outcomes Survey 2016–2020

**DOI:** 10.1016/j.yjcafi.2025.08.008

**Published:** 2026-03-18

**Authors:** HAVISHA PEDAMALLU, SADIYA S. KHAN, CAROL HAYWOOD, ABIGAIL S. BALDRIDGE, KATHLEEN L. GRADY, SARAH CHUZI, PARAG GOYAL, GREGG C. FONAROW, TARA LAGU, FARAZ S. AHMAD

**Affiliations:** 1 Division of Cardiology, Department of Medicine, Northwestern University Feinberg School of Medicine, Chicago, IL; 2 Division of Epidemiology, Department of Preventive Medicine, Northwestern University Feinberg School of Medicine, Chicago, IL; 3 Center for Health Services & Outcomes Research, Institute of Public Health & Medicine, Northwestern University Feinberg School of Medicine, Chicago, IL; 4 Division of Determinants of Health, Department of Medical Social Sciences, Northwestern Feinberg School of Medicine, Chicago, IL; 5 Bluhm Cardiovascular Institute, Northwestern Medicine, Chicago, IL; 6 Division of Cardiac Surgery, Department of Surgery, Northwestern University Feinberg School of Medicine, Chicago, IL; 7 Program for the Care and the Study of the Aging Heart, Division of Cardiology, Department of Medicine, Weill Cornell Medicine, New York. NY; 8 Ahmanson-UCLA Cardiomyopathy Center, Ronald Reagan UCLA Medical Center, Los Angeles, CA; 9 Division of Hospital Medicine, Department of Medicine, Northwestern University Feinberg School of Medicine, Chicago, IL.

**Keywords:** Heart failure, quality of life, comorbidities, activities of daily living, executive functioning

## Introduction

Heart failure (HF) affects over 6.7 million people in the United States and significantly impairs physical, mental and social health. HF experts have often compared HF to cancer, pointing to the similarly high morbidity rates and the need for multidisciplinary care across these populations but with more variable uptake in HF. However, the seriousness of HF may be underestimated by the public.^1^ Although patients with HF experience more burdensome symptoms and similar or worse health-related quality of life (HRQOL) than patients with various cancers,^2,3^ functional impairment has not been compared broadly across these conditions. Functional status, particularly the ability to perform activities of daily living (ADLs), is an important determinant of prognosis, HRQOL and health care needs across diseases. Understanding how functional status in HF compares to that of cancer may help to contextualize the severity of HF, inform prognostic discussions and identify opportunities for targeted interventions that promote independence, improve HRQOL and optimize care delivery.

The objective of this study was to compare functional limitations in adults with HF with those in individuals undergoing treatment for breast, colorectal, lung, or prostate cancer. We hypothesized that patients with HF would have greater functional limitations than those with cancer.

## Methods

The study’s sample included adults aged 65 years and older from the 2016–2020 Medicare Health Outcomes Survey, which is administered by the Centers for Medicare & Medicaid Services and includes data on patient-reported physical and mental health outcomes among Medicare-managed care users in the United States.^4^ We included respondents who self-reported a history of HF or were undergoing active treatment for cancer (lung, colorectal, breast, or prostate).

The primary outcome in this analysis was the ability to perform activities of daily living (ADLs). Basic ADLs, or skills required to manage physical needs and hygiene, were assessed by using the validated Katz Index of Independence in ADLs.^5^ The Katz index (score range = 0–6) for the retrospective assessment of basic ADLs is commonly summarized as severe impairment (0–2), moderate impairment (3–5) and full function (6). Secondary outcomes included examining differences in the Katz index by demographic characteristics and in instrumental ADLs (IADLs), which are more complex skills associated with planning and memory. IADL scores (range = 0–7) were grouped into severe impairment (0–3), moderate impairment (4–6), and full function (7).

We summarized differences by sociodemographics and comorbidities for patients with HF and each subtype of cancer. The *t* test was used to compare ADL scores between HF and each cancer subtype for the overall population and by age groups (65–74 years and 75 years and older). The ^2^ test was used to evaluate differences in the distribution of severe impairment, moderate impairment and full function by condition. All analyses were conducted using SAS (version 9.4) and STATA (version 16.1) software.

## Results

### Baseline Characteristics

A total of 51,400 patients with HF, and 3,805 patients reporting active treatment for lung cancer, 3,830 for colorectal, 12,922 for breast, and 16,783 for prostate cancer were included in this analysis (Table 1). Half (50.2%) of the HF patients were aged 65–74 years. The cancer groups all had a higher proportion of individuals aged 65–74 years compared to the HF group. Patients with HF also had higher rates of comorbidities, including coronary artery disease or history of myocardial infarction (41.9%), depression (32.8%), diabetes (46.6%), hypertension (86.5%), and stroke (19.8%) compared to patients with cancer. Patients with HF had the second-highest prevalence of chronic obstructive pulmonary disease (40.0%), second only to patients with lung cancer (60.0%).

### ADL Scores by Condition

Patients with HF had lower mean Katz index scores (4.16 ± 5.43) and IADL scores (5.43 ± 2.01) compared to the patients with cancer included in this analysis (*P* < 0.001) (Table 2). When limited to patients between 65 and 74 years to account for age-related changes in functional status, patients with HF had lower Katz index scores (4.42 ± 1.76) and IADL (5.86 ± 1.63) scores compared to the other groups (*P* < 0.001) (Table 2). The relationship between Katz scores and IADL scores was largely consistent across other subgroups according to gender, race, marital status, education, and comorbidities (Table 2).

### Distribution of ADL Scores According to Condition

**Fig. 1.**
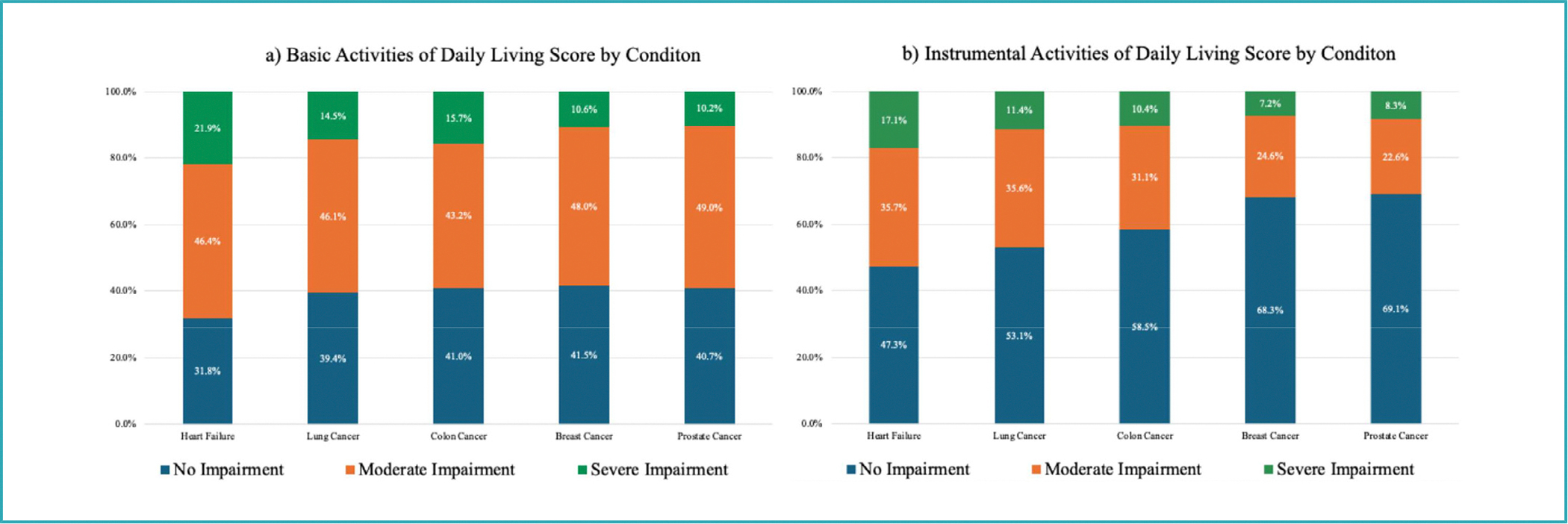
The distribution of the Katz Index of Independence in activities of daily living (ADLs) and instrumental ADLs (IADLs), impairment by condition. Impairment was broken down: severe impairment (0–2), moderate impairment (3–5), and full function (6) for the Katz Index of Independence and into severe impairment (0–3), moderate impairment (4–6), and full function (7) for IADLs.

Of patients with HF, 21.9% had severe impairment according to Katz index scores and 17.1% according to IADL assessments. These numbers were significantly higher than the proportion of severe impairment in patients with lung (Katz: 14.5%; IADL: 11.4%), colorectal (Katz: 15.7%; IADL: 10.4%), breast (Katz: 10.6%; IADL: 7.2%), or prostate (Katz: 10.2%; IADL: 8.3%) cancers (Fig. 1); (all *P* < 0.001). Patients with HF also had the lowest proportion with full function according to the Katz index (31.8%) or IADLs (47.3%).

## Discussion

In this analysis of a large, diverse population of older adults with HF, individuals with HF had greater impairments in both basic and instrumental ADLs than those undergoing treatment for breast, colorectal, lung, or prostate cancer. The high burden of functional disability, coupled with the previously described low HRQOL and high symptom burden of HF, underscores the substantial morbidity associated with the disease.

In oncology, functional capacity is routinely measured and used to inform prognostication and treatment decisions. For example, the Karnofsky Performance Status score, which is widely used in oncology as a standardized way to measure functional status, is strongly correlated with survival in many cancers and is used to inform treatment planning and clinical-trial eligibility.^6^ Further, multidisciplinary care, including palliative care, rehabilitation, nutrition, social work, and survivorship planning, is a standard feature of cancer care, often initiated early after diagnosis.^7^ In contrast, functional capacity in HF is rarely assessed outside of hospitalizations or evaluations for advanced surgical therapies. Similarly, although multidisciplinary care is recommended in HF guidelines, implementation is inconsistent and is typically reserved for later stages of disease. Our findings underscore the need to develop, test and scale care models in HF that prioritize measuring, preserving and improving functional capacity from the time of diagnosis; this approach should mirror the proactive, integrated strategies used in oncology. Such models may improve independence, enhance HRQOL and reduce the health care burden of HF.

The strengths of this study include its national coverage and diverse sample. Although our analysis was limited to individuals with Medicare Advantage, this population still provides meaningful data, because most people with HF are ≥ 65 years old, and Medicare Advantage insures more than half of this group. Limitations include the lack of data regarding disease severity, treatment course and current use of home health services. Self-reported data for HF and cancer ascertainment have the potential for misclassification, but prior publications have used self-reporting to estimate national rates for the prevalence of these conditions.^8^ Another major limitation is our inability to fully isolate the effect of HF from other factors, such as age and comorbidities. However, these characteristics are inherent in the HF population and contribute to their decreased functional status. Rather than diminishing the importance of our findings, this underscores a critical gap and the need for targeted policy interventions to improve outcomes for this vulnerable group.

## Conclusions

We highlight that functional capacity is yet another domain in which HF imposes a greater morbidity burden than cancer. Following successful cancer-care models, the HF community should prioritize early, routine assessment of functional capacity and should expand multidisciplinary care, consistent with guideline recommendations, earlier in the disease course. Further research and advocacy efforts are needed to continue to raise public awareness of the multifaceted and profound public health burden of HF.

## Figures and Tables

**Table 1:** Baseline characteristics of survey respondents by condition

	Heart Failure (n = 51,400)	Lung Cancer (n = 3,805)	Colorectal Cancer (n = 3,830)	Breast Cancer (n = 12,922)	Prostate Cancer (n = 16,783)

Age, years					
65–74	25797 (50.2)	2204 (57.9)	2121 (55.4)	7539 (58.3)	8738 (52.1)
75 and older	25603 (49.8)	1601 (42.1)	1709 (44.6)	5383 (41.7)	8045 (47.9)
Gender					
Female	26936 (47.6)	2024 (53.2)	1924 (50.2)	12741 (98.6)	192 (1.1)
Male	26936 (52.4)	1781 (46.8)	1906 (49.8)	181 (1.4)	16591 (98.9)
Race					
White	39824 (77.5)	3041 (79.9)	2969 (77.5)	10279 (79.5)	13158 (78.4)
Black	7454 (14.5)	490 (12.9)	501 (13.1)	1678 (13.0)	2632 (15.7)
Other	4122 (8.0)	274 (7.2)	360 (9.4)	965 (7.5)	993 (5.9)
Marital Status					
Married	21818 (42.5)	1770 (46.5)	17675 (46.1)	5263 (40.7)	11204 (66.8)
Not married	29582 (57.6)	2035 (57.6)	2065 (53.9)	7659 (59.3)	5579 (33.2)
Education					
< HS or GED	14235 (27.7)	948 (24.9)	963 (25.1)	2240 (17.3)	3031 (18.1)
HS or GED	17387 (33.8)	1393 (36.6)	1255 (32.8)	4385 (33.9)	4453 (26.5)
> HS or GED	19778 (38.5)	1464 (38.5)	1612 (42.1)	6297 (48.7)	9299 (55.4)
Medical History					
CAD or MI	32406 (65.0)	1029 (27.0)	885 (23.1)	1747 (13.5)	3850 (22.9)
COPD	19508 (38.0)	2282 (60.0)	939 (24.5)	2775 (21.5)	3045 (18.1)
Depression	16850 (32.8)	1002 (26.3)	984 (25.7)	3277 (25.4)	2891 (17.2)
Diabetes	23930 (46.6)	1056 (27.8)	1312 (34.3)	3681 (28.5)	4869 (29.0)
Hypertension	44457 (86.5)	2629 (69.1)	2824 (73.7)	9072 (70.2)	11773 (70.2)
Stroke	10158 (19.8)	439 (11.5)	408 (10.7)	989 (7.7)	1519 (9.1)

Values are n (%).

*Other* includes (1) Native American or Alaska Native; (2) Asian Indian; (3) Chinese; (4) Filipino; (5) Japanese; (6) Korean; (7) Vietnamese; (8) Other Asian; (9) Native Hawaiian; (10) Guamanian or Chamorro; (11) Samoan; (12) Other Pacific Islander.

CAD, coronary artery disease; COPD, chronic obstructive pulmonary disease; GED, General Educational Development; HS,high school;, MI, myocardial infarction.

**Table 2:** Average Katz Index and IADL scores among survey respondents by condition

	Heart Failure (n = 51,400)		Lung Cancer (n = 3805)		Colorectal Cancer (n = 3830)		Breast Cancer (n = 12,922)		Prostate Cancer (n = 16,783)	
					
	Katz	IADL	Katz	IADL	Katz	IADL	Katz	IADL	Katz	IADL

Overall	4.16 (1.75)	5.43 (2.01)	4.57 (1.68)*	5.78 (1.70)*	4.55 (1.88)*	5.92 (1.68)*	4.79 (1.52)*	6.22 (1.50)*	4.82 (1.50)*	6.16 (1.56)*
Age, years										
65–74	4.42 (1.76)	5.86 (1.63)	4.66 (1.61)*	5.93 (1.57)	4.72 (1.66)*	6.12 (1.46)*	4.98 (1.39)*	6.44 (1.17)*	4.99 (1.36)*	6.44 (1.23)*
75 and older	3.89 (1.95)	5.00 (2.25)	4.44 (1.76)*	5.59 (1.84)*	4.35 (1.83)*	5.66 (1.89)*	4.52 (1.64)*	5.90 (1.82)*	4.64 (1.61)*	5.87 (1.81)*
Gender										
Female	4.52 (1.76)	5.66 (1.91)	4.70 (1.67)*	5.76 (1.75)*	4.73 (1.70)*	5.95 (1.67)*	4.92 (1.51)*	6.04 (1.64)*	4.83 (1.49)*	5.89 (1.63)*
Male	3.83 (1.91)	5.23 (2.08)	4.45 (1.68)*	5.80 (1.65)*	4.38 (1.78)*	5.88 (1.69)*	4.78 (1.52)*	6.22 (1.50)*	4.32 (1.82)*	6.17 (1.56)*
Race										
White	4.20 (1.85)	5.45 (2.03)	4.61 (1.64)*	5.82 (1.70)*	4.62 (1.70)*	5.96 (1.66)*	4.83 (1.45)*	6.27 (1.47)*	4.88 (1.43)*	6.21 (1.54)*
Black	4.05 (1.92)	5.47 (1.87)	4.42 (1.74)*	5.71 (1.66)*	4.47 (1.83)*	5.95 (1.59)*	4.54 (1.75)*	5.99 (1.58)*	4.63 (1.67)*	6.07 (1.58)*
Other	3.92 (1.99)	5.18 (2.02)	4.37 (1.91)*	5.51 (1.75)*	4.15 (1.96)*	5.49 (1.90)*	4.72 (1.58)*	6.04 (1.60)*	4.56 (1.73)*	5.84 (1.74)*
Marital Status										
Married	4.45 (1.77)	5.57 (1.95)	4.76 (1.62)*	5.71 (1.83)*	4.87 (1.68)*	5.95 (1.69)*	5.06 (1.32)*	6.39 (1.32)*	4.93 (1.41)*	6.17 (1.58)*
Not Married	3.94 (1.92)	5.33 (2.05)	4.41 (1.72)*	5.85 (1.57)*	4.38 (1.78)*	5.89 (1.67)*	4.60 (1.61)*	6.10 (1.60)*	4.60 (1.64)*	6.16 (1.51)*
Education										
< HS or GED	3.79 (2.01)	4.96 (2.19)	4.29 (1.86)*	5.41 (1.96)*	4.25 (1.91)*	5.54 (1.88)*	4.32 (1.83)*	5.70 (1.90)*	4.43 (1.80)*	5.61 (1.93)*
HS or GED	4.19 (1.85)	5.45 (2.02)	4.12 (1.63)*	5.86 (1.63)*	4.55 (1.75)*	5.95 (1.66)*	4.78 (1.52)*	6.20 (1.53)*	4.76 (1.53)*	6.09 (1.61)*
< HS or GED	4.39 (1.76)	5.76 (1.78)	4.71 (1.58)*	5.96 (1.54)*	4.74 (1.61)*	6.11 (1.52)*	4.96 (1.35)*	6.41 (1.26)*	4.98 (1.34)*	6.38 (1.34)*
Comorbidities										
≤ 2	4.65 (1.65)	5.84 (1.83)	4.89 (1.49)*	6.06 (1.53)*	4.87 (1.55)*	6.16 (1.48)*	5.04 (1.31)*	6.44 (1.28)*	5.05 (1.28)*	6.37 (1.35)*
≥ 3	3.77 (1.95)	5.11 (2.09)	4.03 (1.84)*	5.33 (1.87)*	3.75 (1.95)	5.29 (1.96)	3.90 (1.83)*	5.43 (1.90)*	3.94 (1.89)*	5.39 (2.01)*

Values are mean score (standard deviation).

*Other* includes (1) American Indian orAlaska Native; (2) Asian Indian; (3) Chinese; (4) Filipino; (5) Japanese; (6) Korean; (7) Vietnamese; (8) OtherAsian; (9) Native Hawaiian; (10) Guamanian orChamorro; (11) Samoan; (12) Other Pacific Islander.

*t* test performed between heart failure and each cancer subtype

* *P* < 0.05.

GED, General Educational Development; HS, high school; IADL, instrumental activities ofdaily living; Katz, Katz Index score.
